# Atypical Brain Mechanisms of Prediction According to Uncertainty in Autism

**DOI:** 10.3389/fnins.2016.00317

**Published:** 2016-07-08

**Authors:** Alix Thillay, Mathieu Lemaire, Sylvie Roux, Emmanuelle Houy-Durand, Catherine Barthélémy, Robert T. Knight, Aurélie Bidet-Caulet, Frédérique Bonnet-Brilhault

**Affiliations:** ^1^UMR Institut National de la Santé et de la Recherche Médicale U930, Université François-Rabelais de ToursTours, France; ^2^Centre Universitaire de Pédopsychiatrie, CHRU de ToursTours, France; ^3^Helen Wills Neuroscience Institute and the Department of Psychology, University of CaliforniaBerkeley, CA, USA; ^4^Institut National de la Santé et de la Recherche Médicale U1028, Centre National de la Recherche Scientifique UMR S5292, Centre de Recherche en Neurosciences de LyonBron, France

**Keywords:** autism, prediction, uncertainty, ERP, mu oscillations

## Abstract

Resistance to change is often reported in autism and may arise from an inability to predict events in uncertain contexts. Using EEG recorded in 12 adults with autism and age-matched controls performing a visual target detection task, we characterized the influence of a certain context (targets preceded by a predictive sequence of three distinct stimuli) or an uncertain context (random targets) on behavior and electrophysiological markers of predictive processing. During an uncertain context, adults with autism were faster than controls to detect targets. They also had an enhancement in CNV amplitude preceding all random stimuli—indexing enhanced preparatory mechanisms, and an earlier N2 to targets—reflecting faster information processing—compared to controls. During a certain context, both controls and adults with autism presented an increase in P3 amplitude to predictive stimuli—indexing information encoding of the predictive sequence, an enhancement in CNV amplitude preceding predictable targets—corresponding to the deployment of preparatory mechanisms, and an earlier P3 to predictable targets—reflecting efficient prediction building and implementation. These results suggest an efficient extraction of predictive information to generate predictions in both controls and adults with autism during a certain context. However, adults with autism displayed a failure to decrease mu power during motor preparation accompanied by a reduced benefit in reaction times to predictable targets. The data reveal that patients with autism over-anticipate stimuli occurring in an uncertain context, in accord with their sense of being overwhelmed by incoming information. These results suggest that adults with autism cannot flexibly modulate cortical activity according to changing levels of uncertainty.

## Introduction

Autism Spectrum Disorder (ASD) is a pervasive neurodevelopmental disorder characterized by difficulties in social communication and interaction, associated with restricted, repetitive patterns of behavior, interests, or activities (American Psychiatric Association, 2013). The insistence on sameness is a fundamental feature of ASD and is incorporated into diagnostic criteria. Clinical reports of individuals with ASD show that they react in an unusual way (they may feel stressed and anxious) to unpredictable change occurring in their environment. Such a crucial need for stability in individuals with ASD might arise from a dysfunction in the ability to predict events especially in an ever-changing world (Gomot and Wicker, 2012; Pellicano and Burr, 2012; Palmer et al., 2013; Lawson et al., 2014; Van de Cruys et al., 2014). Pathological restricted and repetitive behaviors and interests, rituals, and routines could represent attempts to regulate uncertainty by imposing sameness and order (Gomot and Wicker, 2012; Pellicano and Burr, 2012; Lawson et al., 2014). In addition, social-communication impairments in ASD could be the consequence of difficulties in adapting quickly to the unpredictable social world (Gomot and Wicker, 2012; Lawson et al., 2014; Van de Cruys et al., 2014; Robic et al., 2015). However, to our knowledge, no study has investigated the brain mechanisms of predictive processing in adults with ASD.

Predictive coding formulations of perception propose that expectations in higher brain areas generate top-down predictions that meet bottom-up stimulus signals in lower hierarchical areas (e.g., Friston, 2005). This prediction capacity is essential to efficiently adapt behaviors in an ever-changing world (Bubic et al., 2010). Predictive processing comprises several processes such as the generation of prediction based on encoding of predictive information, and the implementation of prediction via the deployment of both attentional and motor preparatory mechanisms, resulting in facilitated processing of upcoming events, and optimized behaviors indexed by reduced reaction times. In a previous study using a detection task manipulating target predictability (Bidet-Caulet et al., 2012), we defined electrophysiological (EEG) markers of these different stages in typically developing adults. The P3 amplitude to predictive stimuli was found to index predictive information encoding, increase in the Contingent Negative Variation (CNV; pre-stimulus slow ERP) amplitude to reflect the deployment of preparatory mechanisms, decrease in mu power to reflect motor cortex activation, and the P3 latency to predicted target to serve as a measure of the prediction building and implementation (Bidet-Caulet et al., 2012).

While the encoding of explicit predictive non-social cues has not been examined in ASD, some electrophysiological studies have found inconsistent findings, with evidence for atypical preparation in 8–13 year old children (indexed by an increase in CNV amplitude; Tye et al., 2014) or for a preserved preparation in adults (no significant difference in CNV amplitude compared to controls; Strandburg et al., 1993) with ASD without intellectual disability. Reduced motor anticipation has been clinically reported in ASD since the Kanner initial case reports (Kanner, 1943) and recently from a retrospective study (Brisson et al., 2012). Electromyographic studies found substantial anticipation difficulties, reinforcing these clinical observations (Schmitz et al., 2003; Cattaneo et al., 2007). More precisely, an electrophysiological study investigating the theta frequency band in children (which corresponds to the classic mu rhythm recorded in adults) using a bimanual load-lifting task revealed a lack of increased cortical activity of the motor areas before voluntary unloading in the ASD group (Martineau et al., 2004).

Using EEG recorded in 12 adults with autism and age-matched controls performing a visual target detection task, we characterized the influence of a certain context (targets preceded by a 100% predictive sequence of three distinct stimuli), or an uncertain context (random targets) on behavior and electrophysiological markers of predictive processing. Thus, based on this paradigm, we wanted to answer the following questions: (1) do adults with ASD benefit from the predictive information behaviorally (indexed by reduced reaction times)? (2) do the brain mechanisms involved in predictive processing in a certain context are atypical in adults with ASD? (3) do the brain mechanisms involved in predictive processing in an uncertain context are atypical in adults with ASD (4) what steps involved in prediction, such as extraction of predictive information (indexed by an increase in P3 amplitude) required to generate prediction, attentional, and motor preparation mechanisms (reflected by an increase in CNV amplitude and a decrease in mu power) corresponding to the implementation of prediction (indexed by a reduced target-P3 latency) are specifically affected in adults with ASD?

## Materials and methods

### Subjects

Twelve adults with ASD without intellectual disability (10 males and 2 females, 1 left-handed), aged from 18 to 27 years (mean ± Standard Error of the Mean = 21 years, 4 ± 10 months) were recruited from the Child Psychiatry Department specialized in autism, University Hospital of Tours, France. They were diagnosed by expert clinicians according to DSM-IV-TR criteria (American Psychiatric Association, 2000) and using the Autism Diagnostic Observation Schedule-Generic (ADOS-G; Lord et al., 2000) and/or the Autism Diagnostic Interview-Revised (ADI-R; Lord et al., 1994). ASD participants did not present any comorbidity at the time of the study. Intelligence quotients (IQ) were assessed by the Wechsler intelligence scales according to the subjects' ages and developmental levels (Wechsler, 1997, 2005). Intelligence scales provided overall intellectual (mean ± SEM = 101 ± 5), verbal (mean ± SEM = 100 ± 3), and performance quotients (mean ± SEM = 104 ± 7).

Twelve healthy volunteers (mean ± SEM = 21 years, 7 ± 11 months; 10 males and 2 females, 1 left-handed) also participated in the study as control (CTRL) subjects. None of these healthy adults had a previous history of psychiatric or neurological problems and they were not taking any drug. The two groups were matched in age, gender, and handedness. While a full Wechsler was administered to the adults with ASD, two non-verbal subtests (block design and matrix reasoning) of Wechsler intelligence scales were used in the CTRL group. Block design standard scores ranged from 1 to 16 (ASD: 10.8 ± 1.2; CTRL: 11.8 ± 0.8), and matrix reasoning standard scores ranged from 6 to 14 (ASD: 10.1 ± 0.8; CTRL: 10.7 ± 0.4). No significant difference between groups was found on the standard scores obtained from these 2 subtests using randomization tests (*p* > 0.45).

All participants had normal or corrected-to-normal vision. The local ethical committee board (Comité de Protection des Personnes de Tours Ouest-1, France) approved the protocol. Written informed consent was obtained from all participants.

### Stimuli and tasks

**Figure 1:**
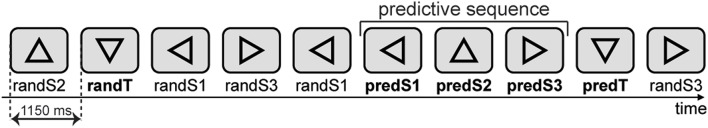
**Stimuli**. A sequence of triangles was centrally presented on a screen. A target could be a random target (randT) preceded by a non-informative context (random sequence of stimuli) or a predictable target (predT) preceded by an informative context, i.e., a three-stimulus predictive sequence (leftward-, upward-, and rightward-facing triangles). Triangles of the predictive sequence are labeled as predS1, predS2, and predS3 stimuli, whereas the corresponding triangles outside the predictive sequence are labeled as randS1, randS2, and randS3, for leftward-, upward-, and rightward-facing triangles, respectively.

Subjects sat in a chair in a sound-attenuated room, 94 cm in front of a 19-inch PC screen. The experimenters and computers delivering the visual stimuli and recording the EEG were located in a separate room. We used a paradigm designed to investigate predictive context processing adopted from Fogelson et al. (2009). Stimuli were presented centrally on a computer screen and subtended 3⋅ of visual angle (Figure 1).

Stimuli consisted of 15% of targets (downward-facing triangle) and 85% of equal amounts of three types of standards: Triangles facing left, upward, or right. A target could be a random target (randT) preceded by an uncertain context (random sequence of stimuli) or a predictable target (predT) preceded by a certain context, i.e., a three-stimulus predictive sequence (leftward-, upward-, and rightward-facing triangles). Triangles of the predictive sequence are labeled as predS1, predS2, and predS3 stimuli, whereas the corresponding triangles outside the predictive sequence are labeled as randS1, randS2, and randS3, for leftward-, upward-, and rightward-facing triangles, respectively. Participants were instructed to press a button with the dominant-hand index finger in response to target stimuli (downward-facing triangles) and to look for the predictive sequence. Before the recording began, subjects performed a first training session to ensure they were able to detect the target accurately. In a second training session, subjects were introduced to the predictive sequence and were aware that it would be 100% predictive of a target, but that targets would also appear randomly throughout the block. Especially for the adults with ASD, training sessions were repeated as many times as necessary to ensure full understanding of the instructions.

In each block (~2.3 min long), a total of 127 stimuli (11 randTs, 28 randS1, 28 randS2, 28 randS3, 8 predTs, 8 predS1, 8 predS2, and 8 predS3) were presented each for 150 ms with an inter-stimulus interval of 1 s. 17 subjects performed 15 blocks, one subject performed 12 blocks, 2 subjects performed 10 blocks, and 4 subjects performed 4–8 blocks due to fatigue. The stimulus presentation and response recordings were controlled using Presentation software (Neurobehavioral Systems, Albany, CA, USA).

### Electroencephalography recording and analysis

EEG was recorded from 64 electrodes using Active Two system (Biosemi, The Netherlands). Vertical eye movements were monitored using electrodes placed above and below the left eye. The signal was recorded with a sampling frequency of 512 Hz and filtered at 0–104 Hz. Data were re-referenced offline to the average potential of the two earlobe electrodes.

EEG analyses were based on results from a previous study (Bidet-Caulet et al., 2012). They were performed on standard and target visual stimuli embedded or not embedded in the predictive sequence. We excluded from further analysis: Trials corresponding to standards after a target, standards before or after a button press, a randS2 standard preceded by a randS1 standard but not followed by a randS3 standard (as it is a potential predS2 standard), missed targets, and targets preceded by less than three standards. Eye-movement artifacts were detected using independent component analysis (ICA) and were selectively removed via the inverse ICA transformation. Only 1 or 2 independent components were removed in each subject to clean the data. In five subjects, the flat or excessively noisy signals at one or two electrodes were replaced by their values interpolated from the remaining electrodes using spherical spline interpolation (Perrin et al., 1989). Trials contaminated with excessive muscular activity in the (−700; 700 ms) time-window relative to stimulus onset were also excluded.

As the number of trials for stimuli embedded in the predictive sequence was lower than for the other stimuli, we equalized the number of trials within each pair of to-be-compared stimuli by random selection, for each participant. On average across participants, we obtained mean ± SEM: 68 ± 4, 83 ± 5, 83 ± 5, and 72 ± 5 clean trials for randS1/predS1, randS2/predS2, randS3/predS3, and randT/predT pairs, respectively, for each participant.

### Event-related potential (ERP) analysis

We averaged single trials, locked to stimulus onset, separately for each of the eight stimulus categories (randS1, randS2, randS3, randT, predS1, predS2, predS3, predT). The resulting event-related potentials (ERPs) were digitally band-pass filtered between 0.5 and 30 Hz to analyze slower components, or between 4 and 30 Hz to extract early and transient responses by filtering out slow and large components (such as the Continent Negative Variation or CNV and P3) that can overlap fast and small responses (Bidet-Caulet et al., 2012). For post-stimulus analysis, ERPs were corrected with a −100 to 0 ms baseline before stimulus onset. For pre-stimulus analysis, ERPs were not baseline corrected. ERP scalp topographies were computed using spherical spline interpolation (Perrin et al., 1989).

### Time-frequency (TF) analysis

We analyzed oscillatory activities by means of a Gaussian Morlet's wavelet decomposition (for details, see Tallon-Baudry and Bertrand, 1999). This method led to a power estimate of both evoked (phase-locked to stimulus onset) and induced (jittering in latency) activities in the TF domain. To distinguish induced from evoked activities (reflecting the frequency content of ERPs), we computed, at each point of the TF domain, the stimulus phase-locking factor from the single-trial TF analysis (Tallon-Baudry et al., 1996). This factor ranges from 0 (uniform phase distribution, i.e., high-latency jitter) to 1 (strict phase-locking to the stimulus). The Rayleigh statistic was used to test for the non-uniformity of phase distribution (Jervis et al., 1983), with a threshold of 0.25 to test non-uniformity with α = 0.05: A phase-locking factor superior to 0.25 indicated a non-uniform phase distribution and the underlying oscillations were considered to be phase-locked to the stimulus. To assess the deployment of oscillatory activities around the stimuli, we analyzed the oscillation on a large time-window (−500; 500 ms) around each type of stimulus. In each group, we applied the same baseline correction to all stimuli by subtracting the mean power between −500 and −250 ms before all S1 onset, in each frequency band. We focused our analysis on the alpha frequency band (8–14 Hz). Since mu rhythm is recorded over the sensorimotor cortex (central electrodes) at the same frequency range than alpha rhythm (Pineda, 2005), we deliberately distinguished mu and alpha oscillations based on the topography. Importantly, no difference was observed between CTRL and ASD on the mean power in the 8–14 Hz band in the −500 to 500 ms time-window around S1.

### Statistical analysis

To assess statistical differences between groups and conditions, we used a repeated-measure analysis of variance (rmANOVA) with group (ASD vs. CTRL) as the between-subject factor and predictability (predictable vs. random) as the within-subject factor.

*Post-hoc* analyses were performed with statistical tests based on permutation or randomization for intra- or inter-group comparisons, respectively (Edgington, 1995). Permutation tests consisted of (1) the random permutation of the 12 pairs (corresponding to the 12 subjects) of values, (2) the sum of squared sums of values in the two obtained samples, and (3) the computation of the difference between these two statistic values. We performed all possible permutations (4096) to obtain an estimate of the distribution of this difference under the null hypothesis. This distribution was then compared to the actual difference between the values in the two conditions. Randomization tests consisted of (1) the random constitution of the two samples to compare, (2) the sum of squared sums of values in the two obtained samples, and (3) the computation of the difference between these two statistic values. We performed 10,000 such randomizations to obtain an estimate of the distribution of this difference under the null hypothesis. This distribution was then compared to the actual difference between the values in the two conditions.

#### Statistical analysis of behavioral data

A button press within the interval of 100–1100 ms after a target onset was considered as a correct response, and a press after a standard was counted as a false alarm (FA). Reaction times (RTs) were computed for correct trials, only. We investigated the benefit in RTs with the predictive context independently of RT to randTs by calculating a RT prediction index [(RT randT-RT predT)/RT randT].

The effect of predictability on the % of hits and RTs was assessed using rmANOVAs. The differences between groups on the % of FAs and the RT prediction index were assessed using randomization tests.

#### Statistical analysis of event-related potentials and oscillatory activities

To investigate predictive processing in adults with ASD, we compared ERPs and oscillatory activities to the same physical stimuli embedded (predictive stimuli) or not embedded (non-predictive stimuli) in the predictive sequence. No difference was predicted and none was observed between predS1 and randS1 as participants did not know at that time if the stimulus was part of the predictive sequence or not.

For statistical analysis, we computed the rmANOVA on the latencies of N1 (105; 230 ms) and P2 (205; 310 ms) peaks at PO4, and on the latencies of P2 (155; 255 ms) and N2 (215; 350 ms) peaks at FCz. For the P3 to targets, we also analyzed the latency and amplitude of the P3 maximum peak at Pz in the (250; 750 ms) time-window.

To go further and beyond peaks and components, we also performed rmANOVAs for each of the 64 electrodes on specific time-windows based on results in previous EEG studies (Fogelson et al., 2009; Bidet-Caulet et al., 2012). To correct for multiple tests, we first calculated a corrected *p*-value across time (e.g., 0.05 divided by the number of tested time-windows) and then an effect was deemed significant if a *p*-value inferior to this threshold was found on at least 4 adjacent electrodes.

To analyze early and transient ERPs, we computed the rmANOVA on the 4–30 Hz band-pass-filtered ERP (pre-stimulus baseline-corrected) amplitude within successive 10 ms time-windows of the (0; 400 ms) time-window relative to stimulus onset. The *p*-value threshold for significance was set to 0.00125.

To analyze pre-stimulus activity, we computed the rmANOVA on the 0.5–30 Hz band-pass-filtered ERP (not baseline-corrected) mean amplitude in the (−150; 0 ms) time-window, corresponding to the CNV (pre-stimulus slow ERP) latencies. We also computed a randomization test on the pre-stimulus activity before all standards (randS) on the mean amplitude in the (−50; 0 ms) time-window to analyze predictive processing within the uncertain context. For ease of reading, we will refer to the CNV component.

We also computed the rmANOVA on the 0.5–30 Hz band-pass-filtered ERP (pre-stimulus baseline-corrected) mean amplitude in the (200; 600 ms) analysis window, corresponding to the P3 latencies. For ease of reading, we will refer to the P3 component.

For oscillatory activities, the rmANOVA was applied to the mean TF energy values within successive 200 ms time-windows regularly shifted by 100 ms to cover the entire analysis time-window (−500; 500 ms). To correct for multiple tests in the time dimension, the *p*-value threshold for significance was set to 0.005. To avoid a possible confound due to inclusion of left-handed participants, time-frequency analysis was run with a sample of right-handed participants only (*n* = 11 for CTRL and ASD). Relation between the RT prediction index and electrophysiological values was assessed using the Spearman rank correlation coefficient.

Results of the rmANOVAs are illustrated on topographical views at a typical latency (usually at the maximum of the difference between conditions). As examples, corresponding ERP or TF time-courses are depicted for a typical electrode showing a significant effect.

The ELAN software package was used for visualization and analysis of EEG, ERP, and TF (Aguera et al., 2011). Custom MATLAB R2010b (MathWorks, Inc) programs were used for rmANOVAs on ERP and TF measures and for the randomization tests. STATISTICA v10 (StatSoft, Inc) software was used for rmANOVAs.

## Results

### Behavioral results

All subjects correctly performed the task (CTRL: 97.0 ± 1.3 and 95.6 ± 1.1%, ASD: 93.3 ± 3.1 and 95.3 ± 1.8%, to randTs and predTs, respectively). No effect of group [*F*_(1, 22)_ = 0.76, *p* = 0.391], nor effect of predictability [*F*_(1, 22)_ = 0.04, *p* = 0.848], nor predictability × group interaction [*F*_(1, 22)_ = 1.13, *p* = 0.300] were found significant for the % of hits. Controls made less FAs (0.24 ± 0.05%) than adults with ASD (0.70 ± 0.19%; *p* = 0.003).

**Figure 2:**
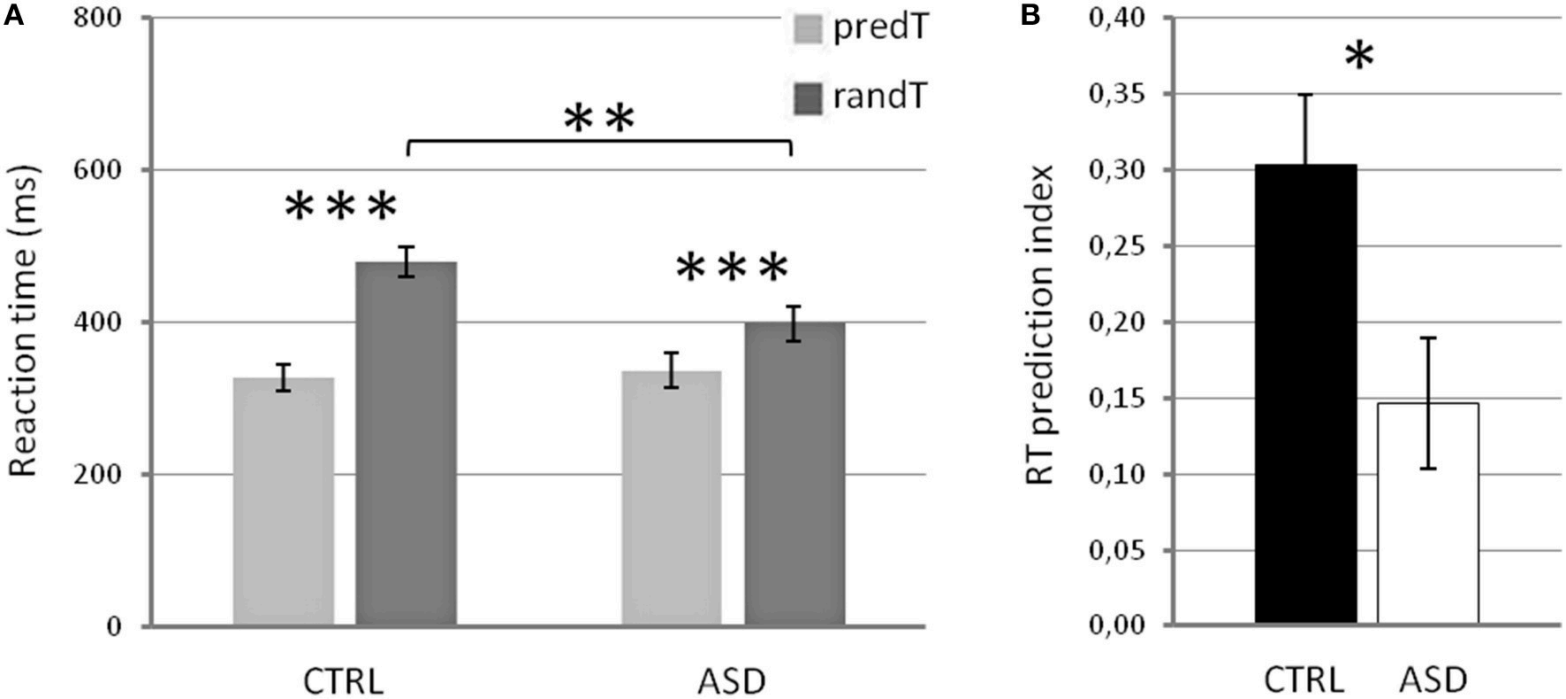
**Reaction times in ms (A) for predictable and random targets, and RT prediction index (B) in controls (CTRL) and adults with ASD**. Error bars: Standard errors of the mean. Significant differences are indicated by asterisks: ^*^p ≤ 0.05, ^**^p ≤ 0.01, ^***^p ≤ 0.001.

Reaction times (RTs) to targets displayed a significant main effect of predictability [*F*_(1, 22)_ = 42.15, *p* < 0.001], a significant predictability × group interaction [*F*_(1, 22)_ = 7.58, *p* = 0.012], but no effect of group [*F*_(1, 22)_ = 2.15, *p* = 0.156; Figure 2]. *Post-hoc* tests showed that, in both groups, RTs to predTs were shorter than those to randTs (*p* ≤ 0.001). Importantly, RTs to randTs were longer in CTRL than in ASD (*p* = 0.012) while no difference was found to predTs (*p* = 0.748). The RT prediction index was also larger in CTRL compared to ASD (*p* = 0.020; Figure 2). In summary, controls present a larger benefit in RTs with the predictive context but are slower to detect randTs than adults with ASD.

### Event-related potential results

#### Early and transient ERPs

No effect was significant on the amplitude of early and transient ERPs in response to targets, S3 or S2, nor on the N1 and P2 latencies at PO4, the P2, and N2 latencies at FCz to S3 or S2 (*p* > 0.061), the target-N1 latency at PO4 (*p* > 0.050), the target-P2 latency at FCz (*p* > 0.068).

A main effect of predictability was found on the target-P2 latency at PO4 [*F*_(1, 22)_ = 6.91, *p* = 0.015], but no predictability × group interaction [*F*_(1, 22)_ = 0.07, *p* = 0.789], nor group effect [*F*_(1, 22)_ = 3.44, *p* = 0.077], with earlier target-P2 latency to randTs than to predTs.

**Figure 3:**
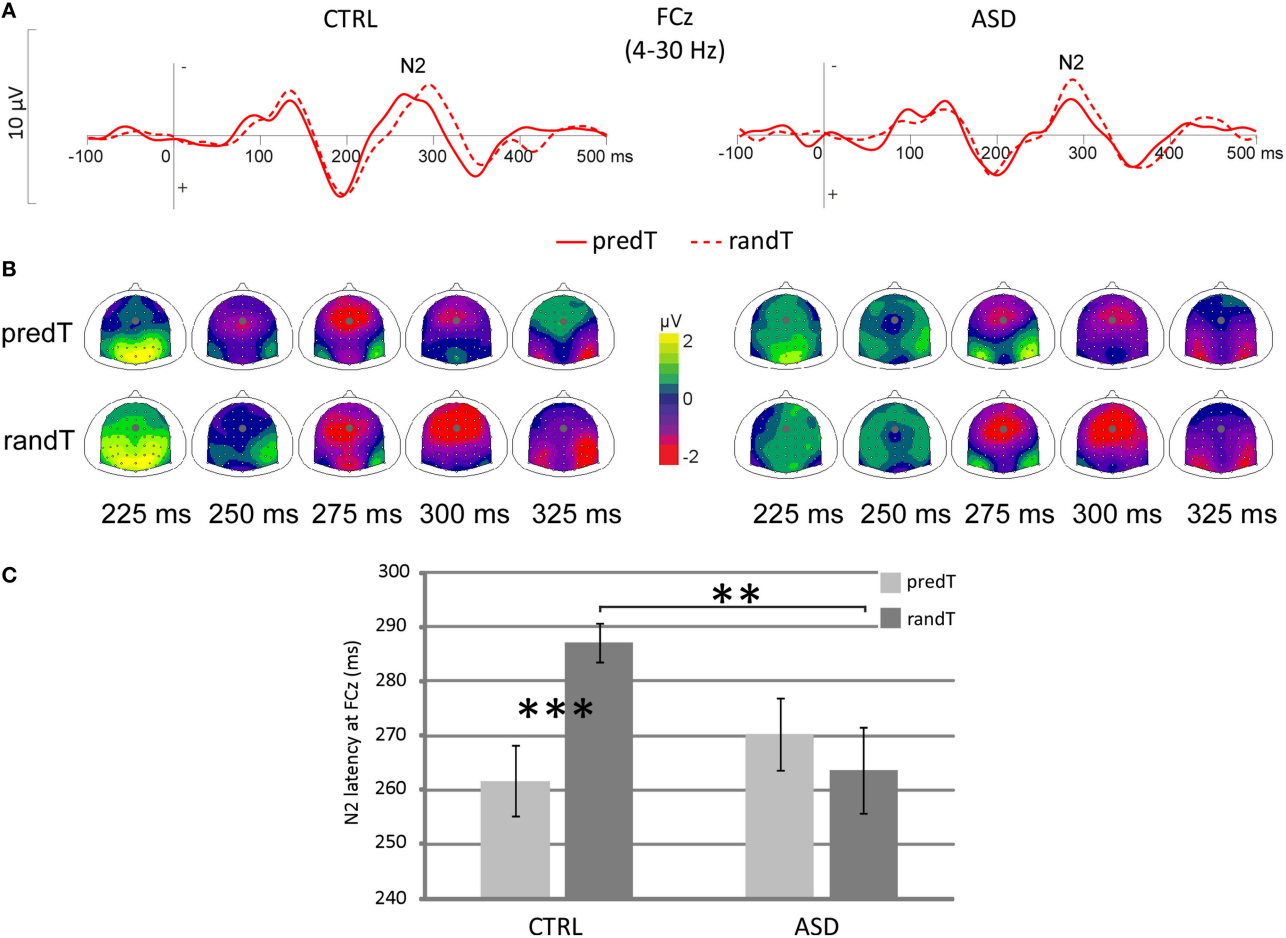
**Effect of predictive context on N2 in controls (CTRL) and adults with ASD**. **(A)** Grand-average ERP waveforms band-pass filtered between 4 and 30 Hz to predTs and randTs (solid and dashed red lines, respectively), at the FCz electrode. **(B)** Scalp topographies (top views) of the N2 to predTs and randTs for controls (CTRL) and adults with ASD. The dots indicate the position of FCz electrode. **(C)** Target-N2 latency at FCz in ms for predTs and randTs in controls (CTRL) and adults with ASD. Error bars = standard errors of the mean. Significant differences are indicated by asterisks: ^**^p ≤ 0.01 ^***^p ≤ 0.001.

A predictability × group interaction was found on the target-N2 latency at FCz [*F*_(1, 22)_ = 7.40, *p* = 0.012], but no predictability effect [*F*_(1, 22)_ = 2.52, *p* = 0.127], nor group effect [*F*_(1, 22)_ = 1.21, *p* = 0.283; Figure 3]. The N2 to predTs was earlier in latency than to randTs in controls only (CTRL: *p* < 0.001; ASD: *p* = 0.440). A reduced target-N2 latency to randTs (*p* = 0.010) but not to predTs (*p* = 0.333; Figure 3C) was found in ASD compared to CTRL.

Only controls displayed a reduction of the target-N2 latency to predTs; whereas adults with ASD showed a reduced target-N2 latency to randTs compared to controls.

#### CNV

**Figure 4:**
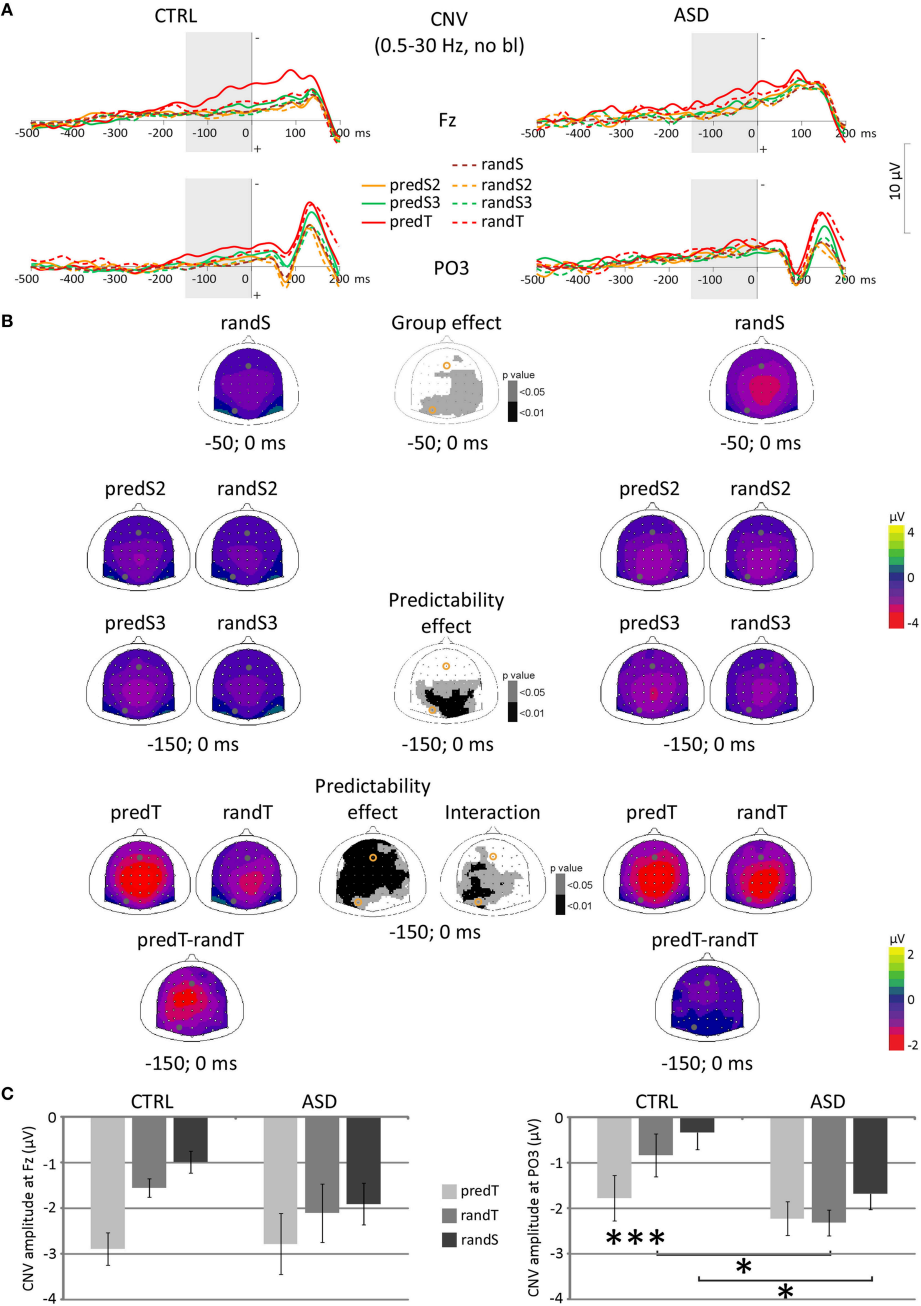
**Effect of predictive context on CNV amplitudes in controls (CTRL) and adults with ASD**. **(A)** Grand-average non-baseline-corrected ERP waveforms band-pass filtered between 0.5 and 30 Hz, at the Fz and PO3 electrodes. **(B)** Scalp topographies (top views) of the mean CNV amplitude for each pair of predictive stimulus and its non-predictive analog, and for the difference between predTs and randTs in the −150–0 ms time-window, and for randS in the −50–0 ms time-window for controls (CTRL) and adults with ASD, and scalp topographies of the *p*-value resulting from the ANOVA. The dots and circles indicate the position of Fz and PO3 electrodes. **(C)** CNV mean amplitude between −150 and 0 ms at Fz and PO3 in μV for predTs, randTs, and between −50 and 0 ms for randS in controls (CTRL) and adults with ASD. Error bars = standard errors of the mean. Significant differences are indicated by asterisks: ^*^p ≤ 0.05, ^***^p ≤ 0.001.

No effect was significant on CNV amplitude preceding S2. A predictability effect, only, was found on CNV amplitude preceding S3, with larger amplitude at parietal electrodes to predS3 [e.g., Pz: *F*_(1, 22)_ = 8.34, *p* = 0.008; Figure 4]. CNV amplitude preceding targets (−150 and 0 ms) displayed a predictability × group interaction at left centro-parietal electrodes [e.g., PO3: *F*_(1, 22)_ = 6.98, *p* = 0.015], an effect of predictability on a large fronto-centro-parietal group of electrodes [e.g., Fz: *F*_(1, 22)_ = 30.05, *p* < 0.001], but no effect of group.

At left centro-parietal electrodes, *post-hoc* tests showed an increased CNV before predTs in comparison to randTs in controls only (e.g., at PO3, CTRL: *p* = 0.022; ASD: *p* = 0.639). Moreover, CNV amplitude to randTs was found larger in ASD than in CTRL (e.g., at PO3, randTs: *p* = 0.014; predTs: *p* = 0.482). Furthermore, randomization test showed an increased CNV before randS at parietal electrodes in ASD compared to CTRL (e.g., at PO3, randS: *p* = 0.018).

In summary, at frontal electrodes, both groups displayed an enhancement of the CNV amplitude before targets with increased predictability. At left centro-parietal electrodes, only controls displayed an enhancement of the CNV amplitude before predTs; whereas adults with ASD showed an increased CNV preceding randTs and randS compared to controls.

#### P3

**Figure 5:**
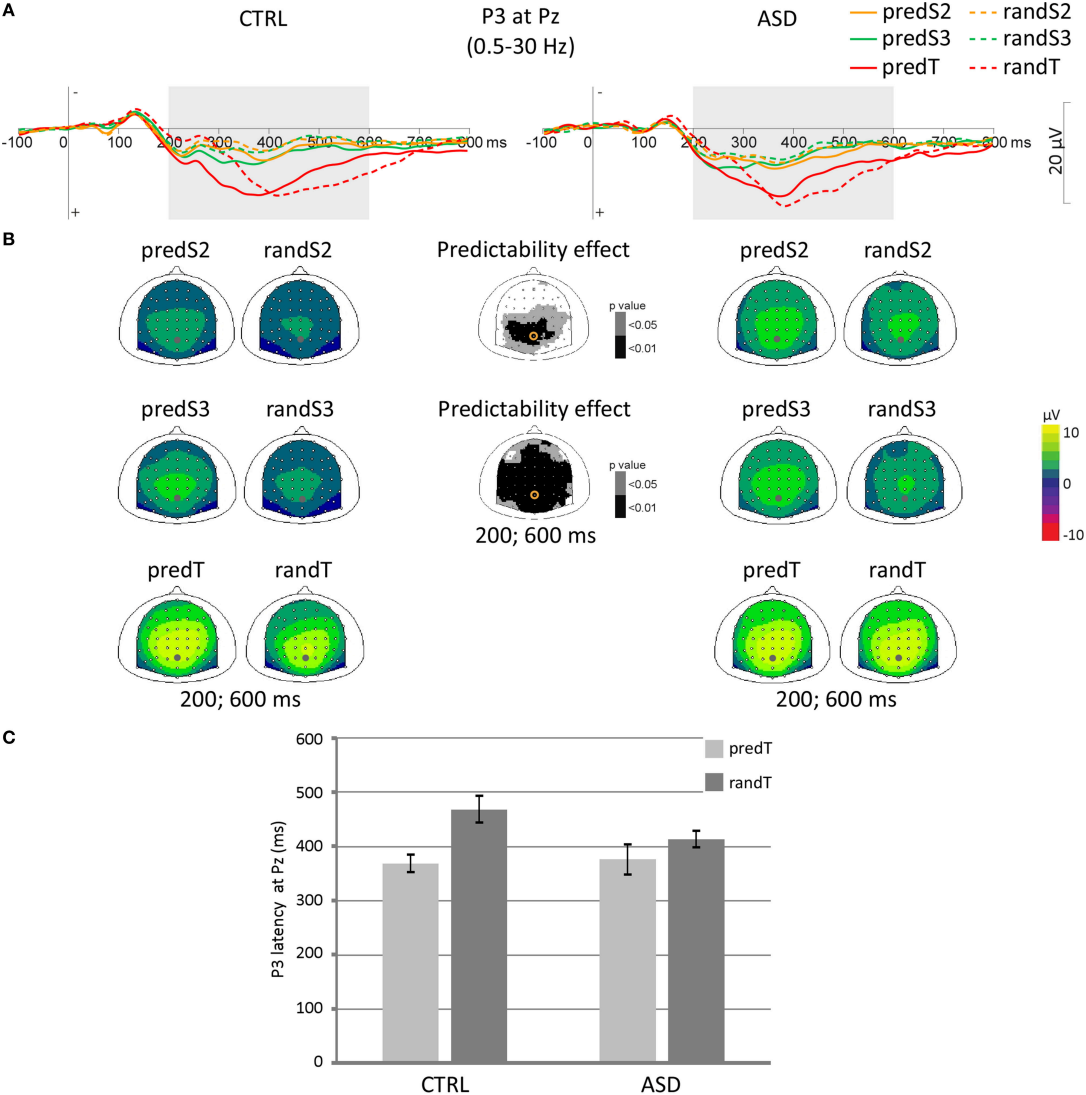
**Effect of predictive context on P3 in controls (CTRL) and adults with ASD**. **(A)** Grand-average ERP waveforms band-pass filtered between 0.5 and 30 Hz, at the Pz electrode. **(B)** Scalp topographies (top views) of the mean P3 ERP for each pair of predictive stimulus and its non-predictive analog in the 200–600 ms time-window for controls (CTRL) and adults with ASD, and scalp topographies of the *p*-value resulting from the ANOVA. The dots and circles indicate the position of the Pz electrode. **(C)** Latency of the P3 maximum in ms at Pz for predicted and random targets in controls (CTRL) and adults with ASD. Error bars = standard errors of the mean.

P3 amplitude to S2 and S3 displayed a predictability effect, only, at centro-parietal electrodes [e.g., Pz: *F*_(1, 22)_ = 10.28, *p* = 0.004; and *F*_(1, 22)_ = 25.73, *p* < 0.001, respectively; Figure 5]. These effects corresponded to an enhancement of the P3 amplitude to standard stimuli with predictive value in both groups.

No effect was found significant on the maximum P3 amplitude at Pz to targets.

A predictability effect was found on the target-P3 latency [*F*_(1, 22)_ = 13.37, *p* = 0.001]. The P3 to predTs was found earlier than to randTs at Pz.

In both groups, P3 amplitude increased throughout the predictive sequence and P3 latency was shortened to predTs.

### Time-frequency results

No effect was found on the 8–14 Hz power before S3 or S2.

#### Left central electrodes

**Figure 6:**
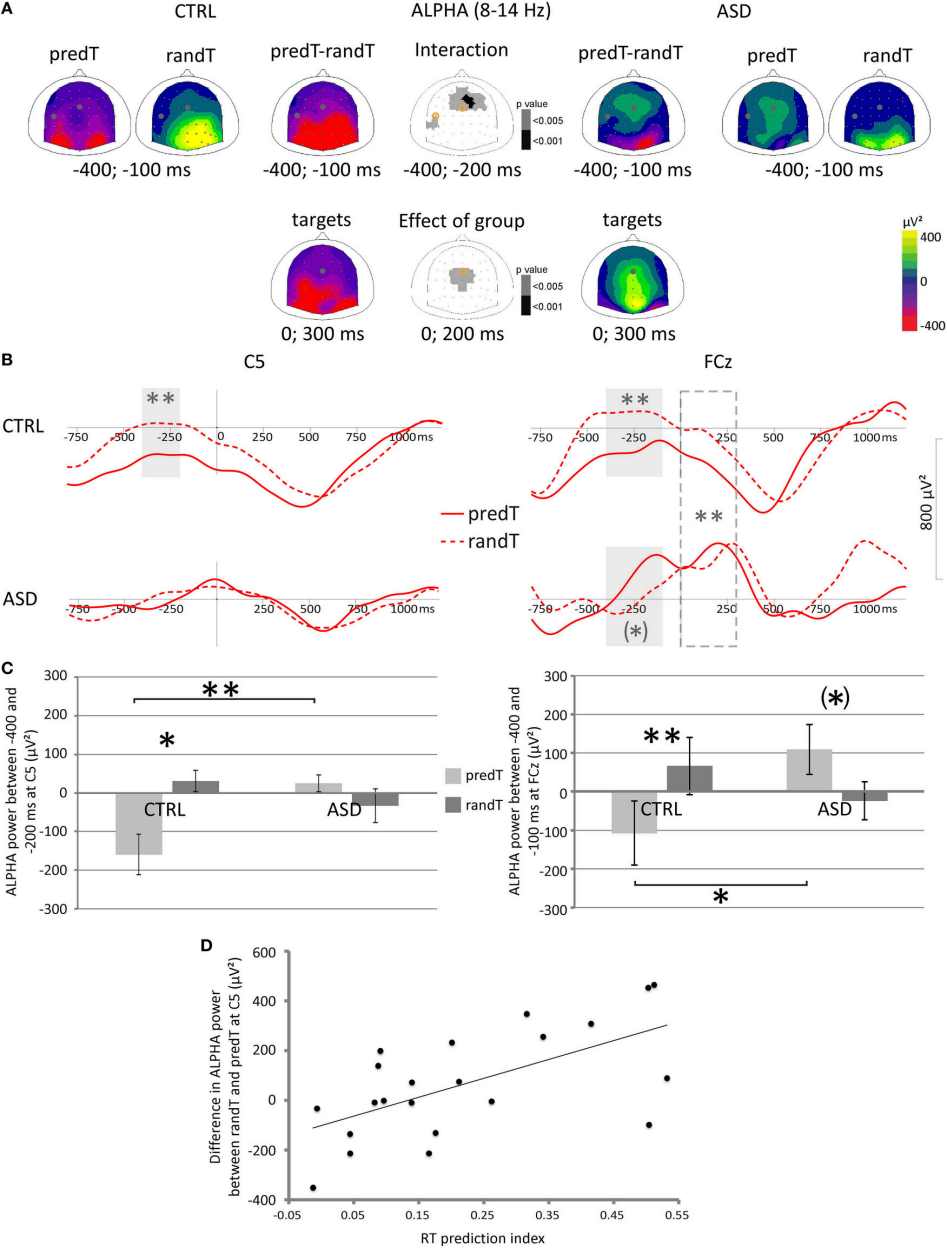
**Effect of predictive context on motor and frontal oscillatory activities in the 8−14 Hz frequency band in controls (CTRL) and adults with ASD**. **(A)** Scalp topographies (top views) of the mean TF power value between −400 and −100 ms and 0 and 300 ms, and of the *p*-value resulting from the ANOVA. The dots and circles indicate the position of the C5 and FCz electrodes. **(B)** Alpha frequency band profiles of TF power at the Fz and C5 electrodes to predTs and randTs (solid and dashed red lines, respectively) in controls (CTRL) and adults with ASD. Time-windows showing a significant difference between the two conditions are indicated by gray bars. Time-window showing a significant difference between the two groups is indicated by dashed gray bar. **(C)** Mean alpha power (μV^2^) between −400 and −200 ms at C5 electrode for predTs and randTs in controls (CTRL) and adults with ASD. Mean alpha power (μV^2^) between −400 and −100 ms at FCz electrode for predTs and randTs in controls (CTRL) and adults with ASD. Error bars = standard errors of the mean. Significant differences are indicated by asterisks: (^*^) *p* = 0.054, ^*^p < 0.05, ^**^p ≤ 0.01 **(D)** Difference in mean alpha power (μV^2^) between randTs and predTs at C5 plotted against the RT prediction index.

A predictability × group interaction was found on the mu power preceding targets between −400 and −200 ms at left central electrodes [e.g., C5: *F*_(1, 20)_ = 10.68, *p* = 0.004; Figure 6]. *Post-hoc* tests showed that only controls displayed a decrease in mu power between −400 and −200 ms before predTs compared to randTs (e.g., C5, CTRL: *p* = 0.015; ASD: *p* = 0.256). Moreover, the decrease in mu power was found larger in CTRL than in ASD between −400 and −200 ms before predTs (e.g., C5: *p* = 0.002), but not before randTs (e.g., C5: *p* = 0.247).

Predictability effect on mu power to targets (difference in mu power between randTs and predTs) at C5 was found correlated with the RT prediction index (*r* = 0.627, *p* = 0.002; Figure 6D). The larger the power reduction, the larger the benefit in reaction time.

#### Fronto-central electrodes

A predictability × group interaction was found on the alpha power preceding targets between −400 and −100 ms at fronto-central electrodes [e.g., FCz: *F*_(1, 20)_ = 11.90, *p* = 0.002; Figure 6]. Controls displayed a decrease in alpha power between −400 and −100 ms before predTs compared to randTs (e.g., FCz: *p* = 0.002); whereas adults with ASD showed a trend for a pre-stimulus alpha increase before predTs compared to randTs (e.g., FCz: *p* = 0.054). The decrease in alpha power was larger in CTRL than in ASD between −400 and −200 ms before predTs (e.g., FCz: ASD > CTRL, *p* = 0.047), but not before randTs (e.g., FCz: *p* = 0.394).

**Figure 7:**
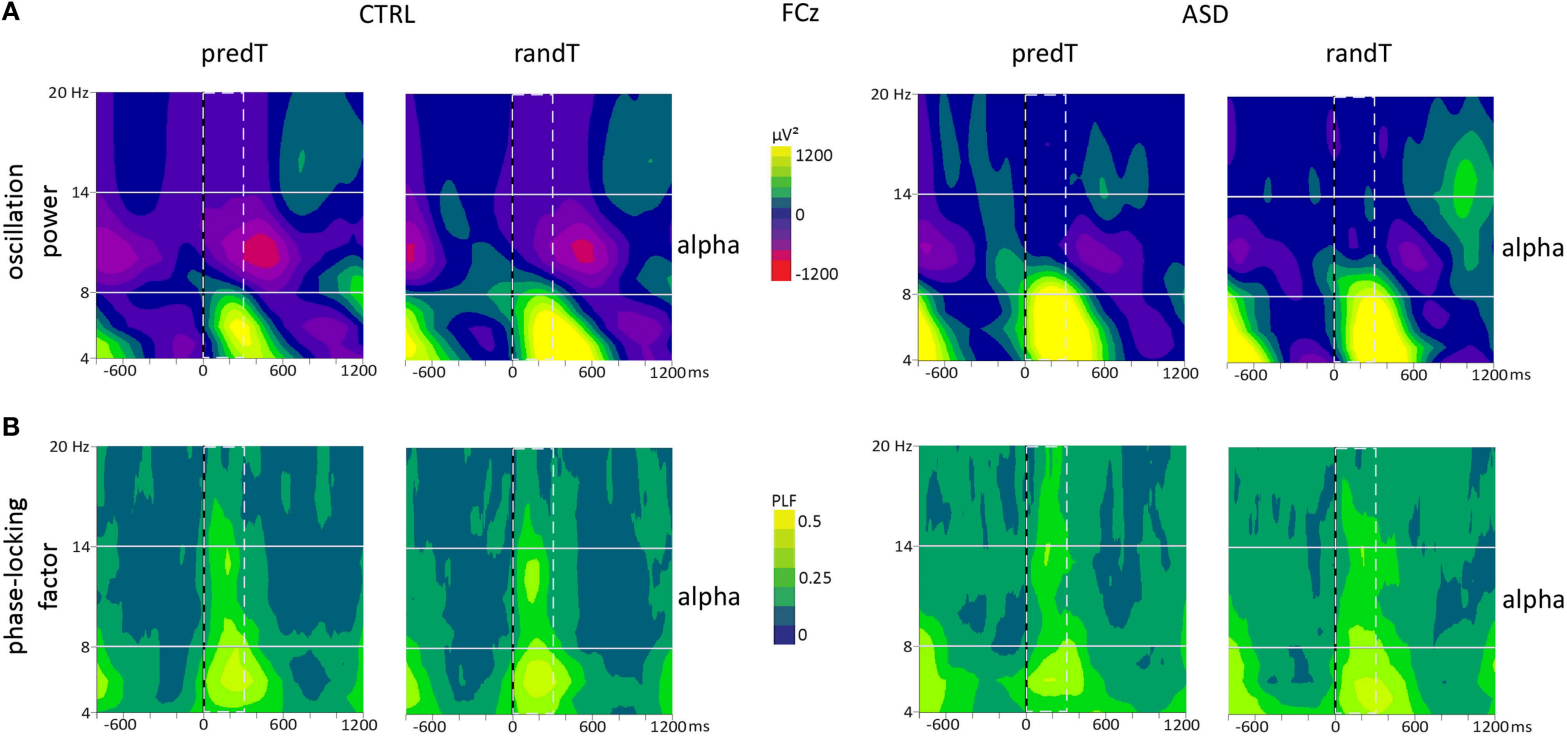
**Effect of predictive context on frontal oscillatory activities in controls (CTRL) and adults with ASD**. **(A)** Grand-average TF plots of the oscillation power and **(B)** phase-locking factor (PLF) at the FCz electrode for predTs and randTs. Alpha (8−14 Hz) frequency band is depicted. Time-window showing a significant difference between the two groups is indicated by dashed bar.

Moreover, a group effect was found on the alpha power between 0 and 300 ms [e.g., FCz: *F*_(1, 20)_ = 14.39, *p* = 0.001]. Controls presented a decrease in alpha power after target onset; whereas adults with ASD showed an increase in alpha power. Analysis of the phase-locking factor indicated an increase in phase-locking to target onset in the alpha band in the same latency range at frontal electrodes in both groups (Figure 7). This increase in phase-locking factor corresponds to the alpha content of the P2 and N2 frontal ERP components.

In summary, adults with ASD did not display a decrease in mu power at left central electrodes before predTs, nor a decrease in alpha power at frontal electrodes after all targets.

## Discussion

During an uncertain context, adults with ASD were faster to detect the target, presented an increased CNV amplitude indexing enhanced preparatory mechanisms, and a shortened N2 latency reflecting faster information processing.

During a certain context, both controls and adults with ASD presented an increased P3 amplitude indexing information encoding of the predictive sequence, an enhanced CNV amplitude corresponding to the deployment of preparatory mechanisms and a reduced target-P3 latency reflecting efficient prediction building and implementation. However, adults with ASD displayed a failure to decrease mu power during motor preparation. This physiological deficit was accompanied by a reduced benefit in reaction times to predictable targets in patients with ASD.

Taken together, the present results provide novel evidence indicating an atypical detection and processing of targets in an uncertain context, coupled with an atypical motor preparation to predictable targets despite a preserved extraction of predictive information.

### CNV and N2: target over-anticipation within an uncertain context

Studies of visual target detection in ASD have reported inconsistent findings, with evidence for equivalent (Tsai et al., 2011) or shorter (Dichter et al., 2009; Maekawa et al., 2011) RTs in response to non-cued target compared to controls. In the present study, adults with ASD were faster than controls to detect the target preceded by a non-informative context with similar overall accuracy. No group differences were found on the visual ERP components, suggesting that targets receive similar degrees of sensory processing in adults with ASD and in controls. Critically, adults with ASD displayed an enhanced CNV before the random standards and targets compared to controls, providing evidence of deployment of atypical increased preparatory mechanisms. In addition, adults with ASD displayed a shortened N2 latency to the random target, suggesting shorter stimulus evaluation (Donchin et al., 1986; Hillyard and Picton, 2011), and response activation time (Smid et al., 1990), supporting faster visual information processing in adults with ASD. The enhanced CNV, the earlier N2 and the shorter reaction times suggest an over-anticipation of stimuli in an uncertain context in adults with ASD. This excessive processing may be counterproductive in daily life and may lead to feelings of sensory overload often reported by individuals with ASD.

### CNV and P3: preserved extraction and use of predictive contextual information

P3 amplitude progressively increased throughout the predictive sequence, i.e., as a function of task relevance and confidence (Sawaki and Katayama, 2006) comparably in controls and ASD subjects. In agreement with a role of the P3 in context-updating (Donchin and Coles, 1988), the present results support the notion that adults with ASD are able, as well as controls, to extract predictive information from the stimulus train.

Adults with ASD displayed a benefit in reaction time with predictive context suggesting that they generate prediction and use it in order to anticipate the predictable target. Moreover, target predictability shortens P3 latency (indicating a shortened duration of stimulus evaluation processing; Kutas et al., 1977; Duncan-Johnson and Kopell, 1981) and enhances CNV amplitude before the predictable targets (reflecting the enhanced recruitment of preparatory mechanisms; Brunia and van Boxtel, 2001) in both groups, confirming that prediction has been implemented.

### Mu oscillations: motor anticipation failure

In accordance with a previous study (Bidet-Caulet et al., 2012), we observed in controls a decrease in mu power before the predictable target onset at left central electrodes, reflecting motor cortex activation prior to execution of the button press (Pfurtscheller and Lopes da Silva, 1999). However, adults with ASD failed to display this mu decrease before the predictable target, suggesting reduced motor preparation. This motor anticipation failure explain why adults with ASD took less advantage from the predictive information compared to controls (smaller RT prediction index).

This result is in accordance with Kanner's first description (Kanner, 1943), and studies on motor anticipatory functions (Schmitz et al., 2003; Martineau et al., 2004) showing major anticipation difficulties.

### Frontal alpha oscillations: atypical frontal mechanisms

Electrophysiological results revealed an increased alpha activity in adults with ASD before the predictable targets over fronto-central regions. This alpha increase may reflect frontal compensation strategies to counteract the lack of motor cortex pre-activation for response execution, or an impairment in integrating prediction with behavior, i.e., an executive dysfunction (Luna et al., 2002).

Moreover, after target onset, adults with ASD presented a phase-locked increase in alpha power at fronto-central electrodes (corresponding to the alpha content of the P2 and N2 frontal ERP components); whereas controls showed a large decrease in alpha power overlapping the phase-locked alpha response. Greater alpha power after both random and predictable targets over the fronto-central regions in adults with ASD may reflect an abnormal inhibition of potential frontal processes needed for executive control during predictive processing, which is consistent with previous findings of atypical executive functions associated with frontal hypo-activation in adults with ASD (Luna et al., 2002).

### Link with predictive coding model

Predictive models of ASD agree about an imbalance of the weight ascribed to bottom-up sensory signals relative to top-down influence of prior information (Brock, 2012; Pellicano and Burr, 2012; Friston et al., 2013; Lawson et al., 2014; Van de Cruys et al., 2014; Skewes et al., 2015) with ASD perception dominated by sensory input. This would result in a tendency to perceive the world in a more veridical way rather than modulated by prior experience (Gomot and Wicker, 2012; Pellicano and Burr, 2012; Lawson et al., 2014; Skewes et al., 2015; Van de Cruys et al., 2014).

According to predictive models, in typically developing individuals, the changing levels of environmental uncertainty determine the assigned weight to prediction errors (Feldman and Friston, 2010; Van de Cruys et al., 2014). In an optimal system, precision in prediction errors (i.e., brain's degree of confidence in the sensory signal) decreases in contexts with higher uncertainty (i.e., when there are no learnable regularities in the environment). The CNV component has been proposed as a proxy for the precision of prediction errors (Feldman and Friston, 2010; Hesselmann et al., 2010) and its amplitude is enhanced with increasing certainty in normal populations. In agreement with this model, the CNV amplitude increased with enhanced predictability of the upcoming stimulus in typically developing participants in the present and previous studies (Bidet-Caulet et al., 2012). Interestingly, we found that adults with ASD generate a larger CNV, compared to controls, in an uncertain context before all random standards and targets. This result suggests that, in the random context, patients with ASD give a high precision to prediction errors as if they were still looking for learnable regularities; whereas typically developing individuals reduce their precision in prediction errors—they sense that there are no learnable regularities. Adults with ASD resist uncertainty and tend to generate higher levels of sensory precision (Van de Cruys et al., 2014). This finding is in line with an inability to flexibly process prediction errors (Palmer et al., 2013; Van de Cruys et al., 2014), and with a failure to attenuate sensory precision.

A limitation of this study is the relatively small sample size. Further, investigations on bigger sample size are needed in order to confirm our results.

We demonstrate that adults with ASD over-anticipate stimuli occurring in an uncertain context. In a certain context, ASD subjects are able to extract predictive information and to use it in order to anticipate the predictable targets. However, the present results may reflect frontal compensation strategies to counteract the lack of automatic motor cortex pre-activation for execution of the motor response. There is a cost to this excessive processing that may be counterproductive in unpredictable and fluctuating situations, such as the social world, leading to stressful reactions, and a sense of overwhelming. Taken together, these results provide evidence that adults with ASD cannot flexibly modulate cortical activity according to changing levels of uncertainty. Moreover, these findings could ultimately contribute to the treatment of adults with ASD without intellectual disability. Further, research is needed in order to build cognitive remediation programs to provide strategies to patients with ASD so that they overcome prediction weaknesses.

## Author contributions

AT has made a substantial contribution to the conception and design, to the acquisition, analysis and interpretation of the data; ML, ED, and CB have made a substantial contribution to the interpretation of the data; SR has made a substantial contribution to the conception and design, and to the acquisition and analysis of data; RK, AC, and FB have made a substantial contribution to the conception and design, and to the analysis and interpretation of the data. All authors have made a substantial contribution to drafting the article or reviewing it critically, have given final approval of the version of the article to be published and have agreed to be accountable for all aspects of the work in ensuring that questions related to the accuracy of integrity of any part of the work are appropriately investigated and resolved.

## Funding

This work was supported by the Fondation Orange (AT), NINDS grant R37NS21135, and the Nielson Corporation (RK). This work was performed within the framework of the LABEX CORTEX (ANR-11-LABX-0042) of Université de Lyon, within the program “Investissements d'Avenir” (ANR-11-IDEX-0007) operated by the French National Research Agency (ANR).

### Conflict of interest statement

The authors declare that the research was conducted in the absence of any commercial or financial relationships that could be construed as a potential conflict of interest.
